# Vitamin D and Disease Perception Outcomes in a Cluster of Patients with High-Disease-Activity Chronic Spontaneous Urticaria

**DOI:** 10.3390/jcm15072717

**Published:** 2026-04-03

**Authors:** Eralda Lekli, Mehmet Hoxha, Maria Bova, Dorian Shkëmbi, Ester Ndreu, Xheini Hajrulla, Elizana Petrela, Etleva Qirko

**Affiliations:** 1 Department of Internal Medicine, Faculty of Medicine, University of Medicine, 1005 Tirana, Albania; mehmethoxha@ymail.com (M.H.);; 2 Salus Hospital Tirana, 1000 Tirana, Albania; 3 Service of Allergology, University Hospital Center Mother Teresa, 1005 Tirana, Albania; 4 Division of Internal Medicine 2, Department of Medicine and Medical Specialties, Antonio Cardarelli Hospital, 80131 Naples, Italy; 5 Department of Public Health, Faculty of Medicine, University of Medicine, 1005 Tirana, Albania

**Keywords:** CSU, vitamin D, disease perception outcomes

## Abstract

**Background/Objectives**: Chronic Spontaneous Urticaria (CSU) is a skin disorder marked by recurrent wheals and itching, with or without angioedema, which can greatly affect the quality of life. Vitamin D has been implicated in the pathophysiology of various immune-mediated conditions, including CSU. The connection between vitamin D levels, patients’ perceived symptoms, and life impact remains unexplored. This study aims to elucidate vitamin D levels and their correlation with perceived disease-related burden in individuals with CSU. **Methods**: PROM-based questionnaires, serum 25(OH)D levels, and BMI were statistically analyzed in high-disease-activity CSU, among hospitalized and outpatients who attended the main tertiary hospital center during a 1-year period. These data were compared with a control group, after obtaining their consent. **Results**: The study included 104 patients, 74 (71.15%) females, mean age 43.17 ± 18.26 years, and 23 controls, 12 (52.17%) females, mean age 44.61 ± 12.77 years. Levels of 25(OH)D were significantly lower in patients compared to controls and in the hospitalized versus the outpatient group (*p* < 0.05). Suboptimal 25(OH)D was found in 94.23% of patients (mean level 18.29 ± 6.74 ng/mL) and 82.61% of controls (mean 24.01 ± 7.44 ng/mL). A BMI > 25 kg/m^2^ was found in 71 (68.3%) patients and 11 (47.83%) controls. Age was not significantly correlated with vitamin D levels. A significant positive correlation was found between vitamin D levels and the perceived bothersomeness score of urticarial elements and pruritus. Age was negatively correlated with perceived bothersomeness of pruritus. Irrespective of 25(OH)D levels, perceived bothersomeness of urticarial elements positively correlated with scores for angioedema, pruritus, and the impact of CSU on life and daily activities. Life and daily activities impact scores were also positively correlated with angioedema and pruritus. **Conclusions**: Suboptimal levels of 25(OH)D were common in CSU patients, especially among hospitalized patients, and were significantly lower compared with controls, suggesting a potential link between low vitamin D status and high disease activity. PROMs did not show a significant association between lower vitamin D levels and worse scores for perceived bothersomeness of urticarial elements, angioedema, pruritus, or impact on life and daily activities.

## 1. Introduction

Vitamin D is recognized not only as a hormone but also as a potent neurosteroid that regulates the processes of neurotransmission, neuroprotection, and immunomodulation [1]. The skin is the main site of vitamin D synthesis, where about 80% of endogenous vitamin D is produced through UVB-induced conversion. After hepatic and renal hydroxylation to its active form, 1,25-dihydroxyvitamin D_3_ (calcitriol), it binds to the vitamin D receptor in cutaneous tissues, regulating proliferation, differentiation, apoptosis, barrier integrity, and immunoregulatory functions [2]. Serum 25(OH)D is the main form of vitamin D circulating in the body; it reflects vitamin D obtained from both skin production and dietary sources [3], and is regarded as the standard clinical indicator of a person’s vitamin D status. 

Deficiency of vitamin D is accepted as 25(OH) vitamin D levels under 20 ng/mL, levels between 21 and 29 ng/mL indicate insufficiency, over 30 ng/mL are sufficient levels (the preferred range is between 40 and 60 ng/mL), and over 150 ng/mL indicate vitamin D intoxication [4]. Maintenance of serum 25(OH)D levels above 30 ng/mL is advised for optimal health outcomes [5].

A recent publication elucidated two main autoimmune pathways that drive CSU: Type I (autoallergic) CSU, mediated by IgE antibodies against autoantigens, and Type IIb CSU, driven by autoantibodies that activate mast cells. Some patients show features of both types, with Type IIb generally being linked to more severe disease and a greater likelihood of coexisting autoimmune conditions [6].

In various autoimmune conditions, patients often show a general decrease in circulating 25(OH)D levels compared to healthy individuals. The clinical significance and underlying mechanisms of this reduction in 25(OH)D during inflammatory diseases are not yet fully understood, but they may be associated with lowered vitamin D binding protein concentrations or an increased rate of 1-α hydroxylation of 25(OH)D [7,8]. However, confounding factors must be considered such as age, high BMI, low physical activity, and baseline comorbidities [7], as each can independently influence serum 25(OH)D levels.

Vitamin D has been implicated in promoting brain health and mood stability, while deficiency is linked to an elevated risk and occurrence of depression [9]. In patients with CSU, low vitamin D levels may worsen symptom perception by influencing mood, fatigue, and coping ability. This could amplify the burden of pruritus and impair daily activities, suggesting that correcting vitamin D deficiency might benefit both inflammatory processes and patient-reported quality of life.

Additionally, vitamin D has been proposed as a contributing factor in the pathogenesis of CSU and a biomarker of disease activity in chronic urticaria [10]. Some studies have reported significantly lower serum vitamin D levels in CSU patients, along with correlations to disease severity [10,11]. However, other studies found no significant difference in vitamin D levels between CSU patients and controls [12,13].

In a recent study, no statistically significant relationship was found between the severity of skin elements scores in the course of CSU and serum vitamin D concentration [14]. Earlier reports from some authors described improvement in urticaria symptoms following vitamin D supplementation [15,16]. Besides the controversy in the reported literature, we found no published study conducted in Albania to investigate a possible correlation of serum 25(OH)D levels with perceived components related to signs, symptoms, and life impact scores using a PROM (Patient-Reported Outcome Measurement) questionnaire in patients with CSU. Therefore, this study addresses a gap in existing research and may contribute to a better understanding and management of CSU patients.

## 2. Materials and Methods

This is a cross-sectional study conducted from September 2024 to September 2025 on Albanian patients with a CSU diagnosis during high disease activity, defined as patients with generalized urticarial elements and pruritus, with or without angioedema, whose symptoms affected daily activity and were not controlled by actual ambulatory treatment when evaluated by the allergist. Participants were categorized into three groups: patients hospitalized for CSU in the Allergology Service at the main university hospital center in Tirana; outpatients consulted for CSU in the Allergology Service at the same hospital; and volunteer controls. All participants underwent serum vitamin D (25(OH)D) level measurement using CLIA (Chemiluminescence Immunoassay) and calculation of body mass index (BMI). The distribution according to 25(OH)D level was categorized as sufficient level (25(OH)D ≥ 30 ng/mL), insufficiency (25(OH)D 20 to <30 ng/mL), and deficiency, with 25(OH)D < 20 ng/mL.

Each patient completed a newly validated four-item questionnaire in the Albanian language, designed to assess their perception of disease-related signs and symptoms scores, and CSU life impact and daily activity impact. The questionnaire, PROMs-based, evaluated four key domains, Q1: bothersomeness scale of urticarial lesions; Q2: bothersomeness scale of angioedema; Q3: bothersomeness scale of pruritus; and Q4: bothersomeness scale of CSU overall impact of the disease on life and daily activities. The questionnaire, originally developed in Albanian, was reviewed by a panel of experts to evaluate content validity. A pilot test with native Albanian-speaking participants ensured clarity, comprehension, and cultural relevance. Reliability was assessed using Cronbach’s alpha of 0.753 to measure internal consistency. Construct validity was evaluated through correlations with related measures, and the final version was refined based on these findings.

**Figure 1:**
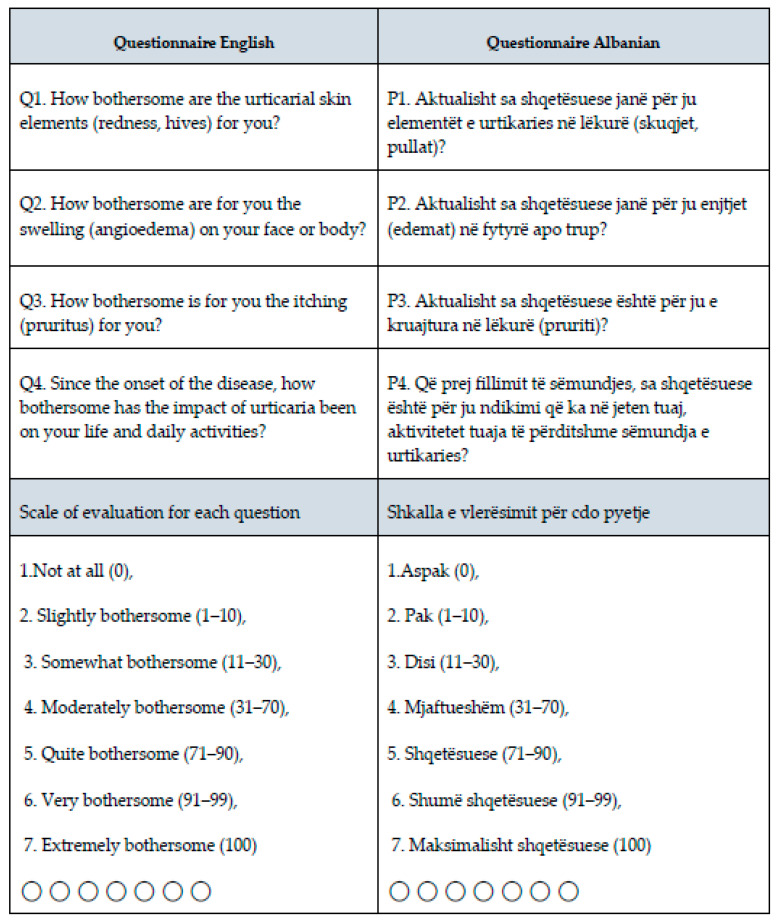
PROMs questionnaire with Q1–Q4 completed from CSU patients; English translation and original Albanian language.

The questionnaire was designed as a hybrid ordinal and numeric scale and organized in seven scales of severity, maintaining both visual analog and metric (Figure 1).

Data were coded in MS Excel 2016 and all statistical analyses were performed on SPSS v.26.0. Continuous variables were described as the means ± standard deviation (SD), whereas categorical variables were described as frequencies and percentages. The Shapiro–Wilk test was used to define the normality distribution in continuous variables, the vitamin D level, and age. Questionnaire responses were treated as ordinal data; therefore, normality testing was not performed for these variables. The Mann–Whitney U test was used to compare differences for continuous variables and the chi-square test for categorical dependent variables. The Kendall’s tau correlation coefficient was used to analyze the correlation of serum vitamin D and other variables. *p* < 0.05 was considered statistically significant and two-tailed tests were performed.

The primary objective was to explore potential correlations between vitamin D levels and the subjective severity scores perception from the questionnaire. Additionally, we aimed to compare vitamin D level-related results, within participant subgroups.

Exclusion criteria included vitamin D supplementation within the past six months, duplicate visits, and the presence of any known psychiatric disorders, as determined from the patients’ anamnestic data, medical records, and medication history. An additional exclusion criterion for the control group was a previous history of urticaria diagnosis.

Study limitations: The control group included volunteers whose vitamin D samples were collected in October. In patients with CSU, samples were collected throughout the year during consultation or hospitalization days. Due to limitations in the number of patients who could undergo vitamin D testing, not all CSU patients who received specialized allergist care during the study period were included in the evaluation.

The study was approved by the Ethic Committee under decision No. 19, dated 11 June 2024, and decision No. 29, dated 29 August 2024. Consent approval was obtained from each participant in the study.

## 3. Results

### 3.1. Demographics

- Age and gender distribution

In this study, a total of 104 participants were included, with a mean age of 43.17 ± 18.26 years. The sample consisted of 30 (28.85%) males and 74 (71.15%) females, reflecting a higher proportion of females (Table 1). The hospitalized group included 43 (41.35%) patients and 61 (58.65%) were in the outpatient group. This sample represents 10.68% (104 out of 973) of CSU patients who received health care services during the study period. The control group included 23 individuals, 12 (52.17%) females with a mean age of 44.61 ± 12.77 years old.

**Figure 2:**
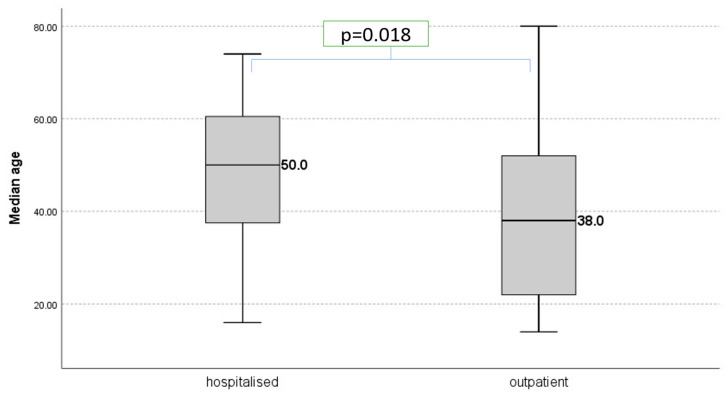
Median age in years in hospitalized and outpatient groups.

The median value of age was significantly higher in the hospitalized group compared with the median age in the outpatient group (respectively 50 vs. 38, *p* = 0.018) as presented in Figure 2. A Mann–Whitney U test did not find a significant difference in age between the control group and the outpatient group (U = 847.00, *p* = 0.144, *n* = 84), nor between the control group and the hospitalized group (U = 426.00, *p* <0.001, *n* = 66). No significant difference in age was found between controls and the study group (U = 1273.00, *p* = 0.630, *n* = 127).

- Body Mass Index

The mean body mass index (BMI) of patients was 26.45 ± 3.78 kg/m^2^. No significant difference in BMI was observed between the hospitalized (25.97 ± 3.48 kg/m^2^) and outpatient (26.8 ± 3.98 kg/m^2^) groups (Mann–Whitney test U = 1474.00, *p* = 0.282, *n* = 104). The mean BMI in the control group was 26.47 ± 4.69 kg/m^2^, with no statistically significant difference between controls and patients (Mann–Whitney test U = 1150.50, *p* = 0.776, *n* = 127).

### 3.2. Vitamin D Level Among Groups

The mean vitamin D levels in patients were 18.29 ± 6.74 ng/mL and 24.01 ± 7.44 ng/mL in controls, which falls below the recommended threshold for optimal vitamin D levels [5].

**Figure 3:**
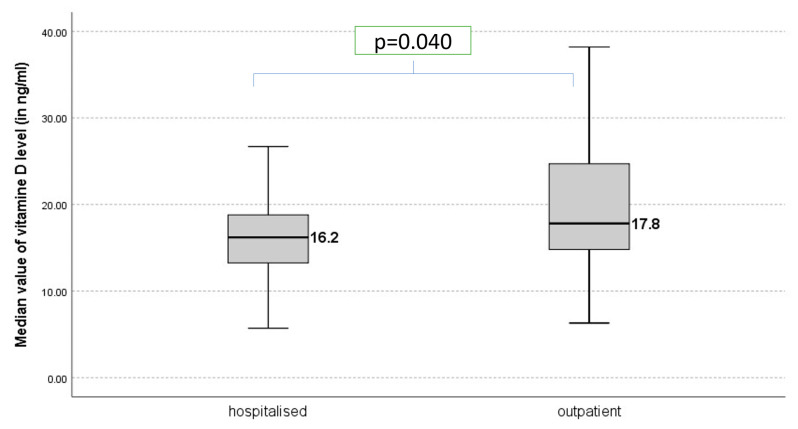
Median value of vitamin D level in hospitalized and outpatient group.

The median value of vitamin D levels was 16.2 ng/mL in the hospitalized group, while in the outpatient group the median value was 17.8 ng/mL, *p* = 0.040 (Figure 3).

The distribution of participants according to 25(OH)D levels showed sufficient levels only in six (5.77%) participants, insufficiency in 26 (25%) participants, and deficiency in 72 (69.23%) participants. In the controls, four (17.39%) participants had sufficient 25(OH)D levels and suboptimal levels were observed in 19 (82.61%) out of 23 participants. A Mann–Whitney U test revealed a statistically significant difference in vitamin D levels between the controls and the study group (U = 1692.00, *p* = 0.002, *n* = 127), as well as between controls and outpatients (U = 922.00, *p* = 0.027, *n* = 84), and between controls and the hospitalized group (U = 770.00, *p* < 0.001, *n* = 66.)

No significant differences in 25(OH)D levels were found between age groups (<50 years and ≥50 years) (Mann–Whitney U-test, *p* > 0.05). Also, Kendall’s tau correlation coefficient showed a non-significant correlation between age and the 25(OH)D level, (*r* = 0.082, *p* = 0.221, *n* = 104) in patients and controls (*r* = 0.125, *p* = 0.411, *n* = 23).

### 3.3. PROMs Result Analysis

PROMs questionnaire results between hospitalized and outpatient groups

All participants responded to a validated, four-question (Q1–Q4), PROMs-based questionnaire to assess the severity scores of bothersomeness perceived related to CSU disease components and the life and daily activities CSU impact. The internal consistency of the questionnaire resulted in a Cronbach’s alpha coefficient of 0.753. The PROMs results were analyzed individually for each component (Q1–Q4) and for each group, in correlation with VIT D levels. Questionnaire mean scores for the total sample ranged from 3.49 ± 1.48 to 4.49 ± 1.48 across Q1–Q4. A comparison between hospitalized patients and outpatient results indicated no statistically significant differences (Table 2) in the bothersomeness scale perception of urticarial elements, pruritus, and the overall impact on life and daily activities of CSU, with Q1 (4.05 ± 1.33 vs. 4.28 ± 1.30; *p* > 0.05), Q3 (4.28 ± 1.47 vs. 4.60 ± 1.48; *p* > 0.05), and Q4 (4.40 ± 0.93 vs. 4.25 ± 1.23; *p* > 0.05). In Q2, which assessed the perceived bothersomeness scale of angioedema (3.86 ± 1.60 vs. 3.13 ± 1.95), the analysis revealed a statistically significant difference between the median values of the hospitalized (Me = 4) and outpatient (Me = 3) groups (*p* = 0.038). Participants in the hospitalized group reported angioedema as more bothersome than the outpatient group. The minimal score of angioedema perception (Not at all (0)) was reported in 19/61 (31.14%) of outpatients, indicating an absence of bothersomeness, and in 6/43 (13.95%) of hospitalized patients.

- PROMs analysis according to VIT D level groups

PROMs results were initially analyzed across two groups according to VIT D levels (Table 3): the deficiency group (G1) with VIT D ≤ 20 ng/mL, had a mean age of 41.64 ± 18.72 years old, and the VIT D insufficient–sufficient (G2) group > 20 ng/mL with a mean age of 46.62 ± 16.99 years old. Both groups were comparable in terms of age distribution and BMI, with no statistically significant difference observed (Mann U test, *p* > 0.05).

- Vitamin D level and Q1–Q4 score correlation analysis

The perceived bothersomeness score of urticarial elements in G2 was significantly higher than in G1 for Q1 (4.63 ± 1.24 vs. 3.99 ± 1.31, *p* = 0.043) and the same was true for pruritus (5.13 ± 1.34 vs. 4.21 ± 1.46, *p* = 0.004). No significant differences were observed between G1 and G2 for angioedema (3.66 ± 2.27 vs. 3.33 ± 1.46, *p* > 0.05) or the impact on life and daily activity (4.69 ± 1.20 vs. 1.32 ± 0.48, *p* > 0.05).

The Kendall’s tau correlation coefficient was calculated to examine the relationship between VIT D, age, and PROMs scores in the sample results. There was a weak but statistically significant positive correlation between VIT D levels and the perceived bothersomeness score of urticarial elements and pruritus using Kendall’s tau correlation coefficient in Q1 *(r = 0*.170, *p* = 0.020) and Q3 (*r* = 0.197, *p* = 0.006). No significant correlation was found between the VIT D level and perceived bothersomeness score of angioedema (Q2), Kendall’s tau correlation coefficient (r=−0.015,p=0.832), and perception scores in Q4 concerning the life impact and daily life activity impact of CSU (*r* = 0.046, *p* = 0.533).

- Age and PROMs results correlation analysis

Results of the Kendall’s tau correlation coefficient showed statistically non-significant correlations between age and the scores in Q1, Q2, and Q4. There was a weak negative correlation between the perceived bothersomeness scores for pruritus (Q3) in CSU and age (r = −0.146,  p=0.043). Correlation coefficients indicated that there was a significant moderate positive correlation between the bothersomeness scale perception of urticarial elements in Q1 and results in Q2 (angioedema) (r = 268, *p* < 0.001); between Q1 and Q3 (pruritus), a significant strong positive correlation (r = 0.616, *p* < 0.001); and between Q1 and Q4 (impact of CSU in life and daily activities), a moderate positive correlation (r = 0.344, *p* < 0.001). A significant moderate positive correlation was observed between perceived scores in Q2 (angioedema) and Q4 (impact of CSU on life and daily activities) with (r = 0.435, *p* < 0.001), and a weak positive correlation with Q3 scores (r = 0.270, *p* < 0.001). Also, analysis indicated that there was a positive correlation between Q3 (pruritus scores) and Q4 (impact of CSU on life and daily activities), regarding severity perception (r = 0.3582, *p* < 0.001).

**Figure 4:**
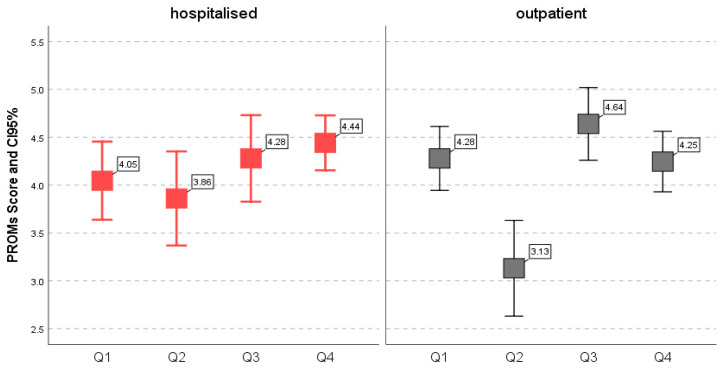
PROMs result in hospitalized and outpatient group according to mean severity score perceived for each individual component Q1–Q4.

Out of 104 patients, 46 (44.23%) reported bothersomeness perception score ≥ 4 (70–100 in the alternative test metric 0–100) regarding the impact of CSU on life and daily activities (Figure 4).

## 4. Discussion

### 4.1. Vitamin D, BMI, and CSU

Several studies suggest an association between CSU and BMI [17,18,19], or metabolic syndrome [17,20]. Chronic urticaria has been linked to increased BMI and a greater likelihood of obesity, suggesting that even at a relatively young age, individuals with CU may already have one or more unrecognized elements of metabolic syndrome [17]. Long-term CSU may be connected to being overweight or obese, and, in turn, having a higher body mass might lead to the development of urticaria symptoms at a later stage [19].

A population-based study including subjects from a primary care setting found a significantly increased risk of CSU in subjects with obesity [18]. However, a French study reported that obesity was not associated with severe CSU [21]. In our sample, patients had a slightly higher BMI (68.5% BMI > 25 kg/m^2^) than controls, but the difference was not statistically significant. This trend is consistent with previous studies associating CSU with being overweight and having metabolic risk factors, suggesting that body mass may contribute to the disease, even if it is not among the primary determinants.

Most of the studies in a recent review suggest that vitamin D deficiency may play a role in the development of obesity in both adults and elderly individuals [22]. A high rate of vitamin D deficiency among obese individuals is well established, likely because the vitamin becomes diluted across larger volumes of fat, as well as in the serum, liver, and muscle tissues [23]. Findings from meta-analyses consistently report an inverse relationship between vitamin D levels and body weight [24]. A consistent association between low serum vitamin D levels and chronic urticaria, particularly chronic spontaneous urticaria, have been reported in several studies [25]. Most participants with CSU in the present study exhibited a suboptimal vitamin D status, with deficiency observed in the majority and only a small proportion having sufficient 25(OH)D levels. Compared with controls, CSU patients had significantly lower vitamin D concentrations, with the lowest levels observed among hospitalized patients. Patients in the hospitalized group also reported angioedema as more bothersome than outpatients, a finding that may reflect greater disease severity. These findings may suggest a possible association between vitamin D deficiency and increased disease activity in CSU, which is consistent with previous reports [10] and supports the proposed immunomodulatory role of vitamin D in inflammatory skin disorders.

However, the high prevalence of suboptimal vitamin D levels in controls indicates that vitamin D deficiency is widespread in the general population and may also be influenced by lifestyle factors such as limited sun exposure and dietary intake.

Although our data appear to support previous observations that vitamin D deficiency may relate to disease activity in CSU, further studies are required to better understand the complex relationship between vitamin D, CSU, and body adiposity.

### 4.2. Vitamin D and Disease Perception

Individuals with CSU face a significant burden, including delayed diagnosis, poor symptom control even with treatment, and considerable effects on their mental, emotional, and social well-being especially when the disease is not controlled [26].

It has been reported that VIT D could serve as a helpful and well-tolerated additional therapy for improving symptoms of depression and anxiety, depending on the patient’s clinical condition and nutritional biomarkers [27]. A large trial found no significant differences in the development or recurrence of depression, clinically meaningful depressive symptoms, or changes in mood scores over a median follow-up of 5.3 years between the VIT D-supplemented and the placebo group [28].

Previous studies have reported that vitamin D supplementation can reduce urticaria symptoms, significantly improving both the severity of CSU and patients’ quality of life [15,16]. Another study did not find any statistically significant relationship between the severity of skin lesion scores in the course of CSU and serum vitamin D concentration [14].

Interestingly, we found a significant positive correlation between vitamin D levels and the perceived bothersomeness of urticarial elements and pruritus. This suggests that, contrary to expectations, higher vitamin D levels may not directly translate into a lower symptom burden in CSU. Age was negatively correlated with the pruritus bothersomeness score, suggesting that younger patients experience itch as more distressing. Also, age and vitamin D were not statistically significantly correlated in our study in either patients or controls.

Importantly, PROMs data demonstrated that the perceived bothersomeness of urticaria, angioedema, and pruritus significantly impacted daily life and daily activities, regardless of vitamin D status. These findings highlight that patient-reported disease burden is multifactorial and not solely dependent on biochemical markers such as 25(OH)D. However, the predominantly suboptimal vitamin D levels observed in the patient sample may limit the direct comparability of these results with those reported in the literature. Angioedema perception scores were significantly higher in hospitalized patients, likely reflecting a greater prevalence of angioedema and more severe disease in this group.

In a considerable proportion of patients (44.23%), the perceived impact of CSU bothersomeness on daily life and daily activities scored ≥4 (70–100), indicating a substantial effect on quality of life.

These results support the utility of the newly validated questionnaire for assessing disease perception in Albanian CSU patients, as no other validated instruments are currently available in the Albanian language.

## 5. Conclusions

Vitamin D deficiency was highly prevalent among CSU patients, particularly in hospitalized individuals, with statistically significantly lower levels compared with controls, supporting a possible association between low serum 25(OH)D levels and high disease activity. No negative correlation was found between 25(OH)D levels and patient-reported outcome measures (PROMs); conversely, a significant positive correlation between vitamin D levels and the perceived bothersomeness of urticarial elements and pruritus was observed. PROMs data did not support the association between lower vitamin D levels and worse scores for the perceived bothersomeness of urticarial elements, angioedema, pruritus, or the impact on life and daily activities.

Regardless of vitamin D levels, scores for urticaria, angioedema, and pruritus were significantly correlated with each other and with the overall impact of CSU on daily life, highlighting that symptom perception strongly influences patients’ daily functioning. Age was not correlated with 25(OH)D levels, but it was negatively correlated with the pruritus bothersomeness score, suggesting that younger patients experience itch as more distressing. The PROMs questionnaire is a reliable and useful tool for assessing symptom perception and disease impact in Albanian-speaking CSU patients, supporting its use for individualized disease management.

## Figures and Tables

**Table 1:** Summary of demographic data and PROMs results.

Category	Total *n* = 104 (%)	Hospitalized(H) *n* = 43 (%)	Outpatients(O) *n* = 61 (%)	*p*-Value
Males	30 (28.85)	10 (23.26)	20 (32.79)	0.380 ^‡^
Females	74 (71.15)	33 (76.74)	41 (67.21)	
Age (years)	43.17 ± 18.26 [Me = 42.5; IQR: 26.0–56.0]	48.05 ± 16.11 [Me = 50.0; IQR: 37.0–61.0]	39.74 ± 19.03 [Me = 38.0; IQR: 22.0–52.5]	0.018 *
BMI (kg/m^2^)	26.45 ± 3.78	25.97 ± 3.48	26.79 ± 3.98	0.282 *
Vitamin D 25(OH)D ng/mL	18.29 ± 6.74 [Me = 16.7; IQR: 13.9–23.6]	16.62 ± 5.47 [Me = 16.2; IQR: 13.2–18.9]	19.47 ± 7.31 [Me = 17.8; IQR: 14.8–24.8]	0.040 *

IQR—interquartile range; Me—median. Percentages are calculated in colons, ^‡^—chi-square test, *—Mann–Whitney U-test.

**Table 2:** PROMs results.

Category	Total *n* = 104	Hospitalized (H) *n* = 43		Outpatients (O) *n* = 61		Mann–Whitney Test Value	*p*-Value
	Mean ± SD	Median (IQR)	Mean ± SD	Median (IQR)	Mean ± SD		
Question 1 (Q1)	4.18 ± 1.31	4.0 {3–5}	4.05 ± 1.33	4.0 {4–5}	4.28 ± 1.3	1472	0.271
Question 2 (Q2)	3.49 ± 1.48	4.0 {3–5}	3.86 ± 1.6	3.0 {1–5}	3.13 ± 1.95	1003	0.038
Question 3 (Q3)	4.49 ± 1.48	4.0 {3–6}	4.28 ± 1.47	5.0 {4–6}	4.6 ± 1.48	1521	0.158
Question 4 (Q4)	4.32 ± 1.11	4.0 {4–5}	4.4 ± 0.93	4.0 {3–5}	4.25 ± 1.23	1173	0.341

SD—standard deviation, IQR—interquartile range.

**Table 3:** Results analyzed into two groups according to VIT D levels.

	No	VIT D (ng/mL)	Age (Years)	BMI (kg/m^2^)	Q1	Q2	Q3	Q4
VIT D ≤ 20 ng/mL	72	14.5 ± 3.32	41.64 ± 18.72	26.1 ± 3.88	3.99 ± 1.31	3.33 ± 1.63	4.21 ± 1.46	4.17 ± 1.05
VIT D > 20 ng/mL	32	26.84 ± 4.07	46.62 ± 16.99	27.26 ± 3.49	4.63 ± 1.24	3.66 ± 2.27	5.13 ± 1.34	4.69 ± 1.2
*p* value			*p* = 0.200	*p* = 0.153	*p* = 0.021	*p* = 0.41	*p* = 0.003	*p* = 0.028

## Data Availability

The datasets presented in this article are not readily available because the data are part of an ongoing study. Requests to access the datasets should be directed to authors and made available upon reasonable request.

## Abbreviations

The following abbreviations are used in this manuscript:

VIT D	Vitamin D
CSU	Chronic Spontaneous Urticaria
UVB	Ultraviolet B
BMI	Body Mass Index
PROMs	Patient-Reported Outcome Measurements
25(OH)D	25-hydroxyvitamin D
